# Lipocalin-2 as an Infection-Related Biomarker to Predict Clinical Outcome in Ischemic Stroke

**DOI:** 10.1371/journal.pone.0154797

**Published:** 2016-05-06

**Authors:** Sonja Hochmeister, Odilo Engel, Milena Z. Adzemovic, Thomas Pekar, Paul Kendlbacher, Manuel Zeitelhofer, Michaela Haindl, Andreas Meisel, Franz Fazekas, Thomas Seifert-Held

**Affiliations:** 1 Department of Neurology, Medical University of Graz, Graz, Austria; 2 Department of Experimental Neurology, Charité Universitaetsmedizin Berlin, Berlin, Germany; 3 Neuroimmunology Unit, Department of Clinical Neuroscience, Center for Molecular Medicine, Karolinska Institutet, Stockholm, Sweden; 4 University of Applied Sciences Wiener Neustadt, Wiener Neustadt, Austria; 5 Department of Neurosurgery, Charité Universitaetsmedizin Berlin, Berlin, Germany; 6 Division of Vascular Biology, Department of Medical Biochemistry and Biophysics, Karolinska Institutet, Stockholm, Sweden; INSERM U894, FRANCE

## Abstract

**Objectives:**

From previous data in animal models of cerebral ischemia, lipocalin-2 (LCN2), a protein related to neutrophil function and cellular iron homeostasis, is supposed to have a value as a biomarker in ischemic stroke patients. Therefore, we examined LCN2 expression in the ischemic brain in an animal model and measured plasma levels of LCN2 in ischemic stroke patients.

**Methods:**

In the mouse model of transient middle cerebral artery occlusion (tMCAO), LCN2 expression in the brain was analyzed by immunohistochemistry and correlated to cellular nonheme iron deposition up to 42 days after tMCAO. In human stroke patients, plasma levels of LCN2 were determined one week after ischemic stroke. In addition to established predictive parameters such as age, National Institutes of Health Stroke Scale and thrombolytic therapy, LCN2 was included into linear logistic regression modeling to predict clinical outcome at 90 days after stroke.

**Results:**

Immunohistochemistry revealed expression of LCN2 in the mouse brain already at one day following tMCAO, and the amount of LCN2 subsequently increased with a maximum at 2 weeks after tMCAO. Accumulation of cellular nonheme iron was detectable one week post tMCAO and continued to increase. In ischemic stroke patients, higher plasma levels of LCN2 were associated with a worse clinical outcome at 90 days and with the occurrence of post-stroke infections.

**Conclusions:**

LCN2 is expressed in the ischemic brain after temporary experimental ischemia and paralleled by the accumulation of cellular nonheme iron. Plasma levels of LCN2 measured in patients one week after ischemic stroke contribute to the prediction of clinical outcome at 90 days and reflect the systemic response to post-stroke infections.

## Introduction

Ischemic stroke is one of the leading causes of death and disability and utilises huge amount of health care expenses [1]. Brain ischemia elicits systemic immunodepression that promotes post-stroke infections. These occur in approximately one third of all stroke patients and impair long-term recovery [2,3]. Lipocalin-2 (LCN2), also called neutrophil gelatinase-associated lipocalin or 24p3, is a crucial component of neutrophils [4–7] and expressed in a wide range of hematogenous and non-hematogenous cells and tissues [8]. LCN2 participates in innate immune responses, impacts on cell proliferation and differentiation [9], and regulates iron homeostasis. Iron-loaded LCN2 enters cells via binding to the receptors 24p3R and LRP2/megalin [10,11]. Iron-free LCN2 chelates intracellular iron and transfers it to the extracellular space [10].

LCN2 has been established as a biomarker of acute kidney injury and a prognostic factor in chronic kidney disease [12]. It was shown to be associated with mortality from cardiovascular disease independent of traditional risk factors and kidney function [13]. Elevated circulating levels were found in patients with transient ischemic attack and acute ischemic stroke [14]. In a mixed cohort of ischemic and hemorrhagic stroke patients, raised LCN2 levels in the peripheral blood obtained within 24 hours from symptom onset were associated with 6-month mortality [15]. In animal models of peripheral lipopolysaccharide administration to induce a septic inflammation, the protein was upregulated in choroidal epithelia, endothelial cells, astrocytes and microglia [16,17]. In animal models of inflammatory brain injury [18–20], intracerebral hemorrhage [21] and ischemic stroke [22,23], LCN2 upregulation occurred predominantly in astrocytes and, to a lesser extent, in neurons. The significance of this finding is currently under debate. Some authors conclude a role in promoting the migration of astrocytes, microglia and neurons by induction of chemokine expression [24–26]. Others have proposed a relation of LCN2 expression with the induction of astrogliosis and neuronal cell death [23,26,27]. These conflicting results may be derived from different models and species used and different doses of LCN2 applied in tissue experiments [28]. The expression of LCN2 in *in vitro* cultured glial cells and in animal models has focussed attention to its putative use as a biomarker of CNS injury including ischemic stroke [29]. In our study, we examined peripheral and central expression of LCN2 in a mouse model of transient middle cerebral artery occlusion (tMCAO). Furthermore, plasma levels of LCN2 were measured in patients one week after ischemic stroke and analyzed for a correlation to post-stroke infections and clinical outcome at 90 days.

## Materials and Methods

### Ethics statement

The study was approved by the institutional review board of the Medical University of Graz (IRB00002556). Written informed consent was obtained from all participants. For patients with impaired consciousness or aphasia, written informed consent was obtained when these patients regained the ability to communicate and were oriented to self and time. No surrogate consent procedure was applied. Animal experiments were performed in accordance with the European directive on the protection of animals used for scientific purposes and all other applicable regulations and approved by the relevant authority, Landesamt fuer Gesundheit und Soziales, Berlin, Germany.

### Animal model of temporary middle cerebral artery occlusion (tMCAO)

Male SPF C57Bl6/J mice (Charles River Laboratories, Sulzfeld, Germany) were housed in cages lined with chip bedding and environmental enrichment (mouse tunnel and igloo; Plexx B.V., Elst, The Netherlands) on a 12 h light/dark cycle (change 7 o’ clock) with ad libitum access to food (standard chow) and water. At the time of the experiment, mice were 11–14 weeks old. Cerebral ischemia was induced as described previously [30]. Briefly, a silicone coated filament was introduced into the internal carotid artery, pushed forward to occlude the middle cerebral artery at its origin and removed after 60 min. Isoflurane (Abott, Wiesbaden, Germany) in a 1:2 mixture oxygen/nitrous oxide was used for anaesthesia, and body temperature was maintained throughout the whole procedure by a feedback controlled heating pad during the operation. Animals were subsequently placed in heated cages (30°C) for a recovery period of 2 hours before returning to their home cages. Animals were allowed free access to food and water. Beginning one day prior to induction of cerebral ischemia until day 6 post tMCAO, mice received preventive antibiotic treatment with marbofloxacin i.p. 5 mg/kg per day.

Ischemic lesion volumes were measured on day 1 after tMCAO by T2 weighted magnetic resonance imaging on a 7T scanner (Pharmascan 70/16 AS, Bruker Biospin, Ettlingen, Germany). Delineable hyperintense lesion volume was determined on 20 consecutive coronal slices with 500μm thickness using Analyze 5.0 (AnalyzeDirect, Overland Park, KS, USA). Lesion volume was edema-corrected [31]. Mice with a lesion volume <25 mm^2^ were excluded from further evaluation.

Animals were sacrificed on day 1 (n = 12), day 7 (n = 11), day 14 (n = 12), day 28 (n = 7) and day 42 (n = 7) post tMCAO under high doses of ketamin/xylazin anaethesia by transcardial perfusion with 4% paraformaldehyde (PFA). Brains were removed and postfixed in 4% PFA for 24 hours. Paraffin embedded sections were examined by immunohistochemistry. Ischemic lesions were delineated by reduced anti-MAP2 immunostaining as reported previously (abcam, Cat.-No. ab32454) [32]. Other primary antibodies were applied as follows: anti-MAC3 (BD Biosciences, Cat.-No. 553322), anti-LCN2 (Sino Biological, Cat.-No. 50060-RP02), anti-neuronal nuclei (NeuN; Millipore, Cat.-No. MAB377), anti-glial fibrillary acidic protein (GFAP; Thermo Scientific, Fremont, CA; Cat.-No. MS-1376). Bound primary antibody was detected with a biotin-avidin technique using 3,3’-diaminobenzidine-tetrahydrochloride (DAB; Sigma, St. Louis; MO, USA) as chromogen. Control sections were incubated in the absence of primary antibody. Double staining for LCN2/NeuN was performed with antibodies from different species and detection by DAB and histogreen (Linaris, Wertheim, Germany). Quantification of labeled cells in the striatum of ischemic hemispheres and respective contralateral regions was performed by an ocular morphometric grid under a 200x objective. Values were transformed to cells/mm^2^.

For ferrous nonheme iron, a DAB-enhanced Turnbull blue reaction procedure was applied as described elsewhere [33]. Briefly, sections were deparaffinised and incubated in ammonium sulphide 2% in distilled water (Merck, Darmstadt, Germany) for 90 min. After washing, sections were treated with an aqueous solution of 10% potassium ferricyanide (Merck, Darmstadt, Germany) in 0.5% HCl. Endogenous peroxidase was blocked in methanol using 0.01 M sodium azide and 0.3% hydrogen peroxide for 1 h. Tissue iron was visualized by 20 min incubation in 0.025% DAB and 0.0005% hydrogen peroxide in 0.1 M PBS.

Serum was obtained from mice 7 days post tMCAO (n = 15) and from a group of naïve control mice (n = 11) and stored at -80°C for analysis. LCN2 levels in mouse serum were analyzed by ELISA according to the manufacturer’s instructions (Kit 042; BioPorto Diagnostics, Gentofte, Denmark).

### Patients

Patients with a diagnosis of ischemic stroke according to clinical examination and brain imaging (computerized tomography or magnetic resonance imaging) were eligible when they had a National Institutes of Health Stroke Scale (NIHSS) of more than 3 on admission and a modified Rankin Scale (mRS) of 0 or 1 before symptom onset. The NIHSS was obtained on admission by board certified neurologists. The mRS was obtained 90 days post stroke by telephone interviews with the patients or their caregivers [34]. Stroke was classified according to the Oxfordshire Community Stroke Project (OCSP) [35] and the Causative Classification of Stroke System (CCS) [36]. Patients were excluded from the study if their neurological symptoms persisted for less than 24 hours and if there was already evidence for any infectious disease including an abnormal leukocyte count on admission. Patients were not eligible when they had major surgery or transfusion of blood components in a timeframe of 3 weeks prior to the stroke. Further exclusion criteria were applied as follows: acute renal failure, chronic hemodialysis, congestive heart failure NYHA III/IV, active malignancy, immunosuppressive treatment. Cerebrovascular risk factors were identified as defined by preadmission history or the need for medication at discharge: hypertension, hypercholesterolaemia, and diabetes mellitus. Atrial fibrillation was diagnosed either by history, an electrocardiogram (ECG) on admission, or Holter-ECG during the hospital stay. None of the patients involved in the study suffered from a myocardial infarction. Post-stroke infections were defined as infections which became evident by clinical, radiological or laboratory means after the onset of acute stroke and required antibiotic treatment. No preventive antibiotics were given. Clinical care was performed according to the guidelines of the European Stroke Organization. Venous blood was drawn at 8.00 a.m. one week after acute stroke onset (median 7 days, range 5–9). Lithium-heparin plasma samples were stored at -80°C for further analysis. LCN2 levels in patient plasma were analyzed by ELISA according to the manufacturer’s instructions (Kit 036CE; BioPorto Diagnostics, Gentofte, Denmark). C-reactive protein (CRP) levels and the estimated glomerular filtration rate (eGFR), determined by the Modification of Diet in Renal Disease formula [37], were obtained by standard laboratory procedures. One patient received atorvastatin at the time of blood sampling which was previously shown to decrease plasma levels of LCN2 [38].

### Statistical analysis

Student’s t-test, Mann-Whitney’s U-test, the Chi-square test or Fisher’s exact test, and Spearman’s rank order correlation were applied for two-group comparisons. The level of significance was set at a p-value of less than 0.05. A stepwise linear logistic regression model was used for analyzing variables to predict clinical outcome. Variables with p-values less than 0.1 were included in an initial predictive model. Backwards elimination logistic regression was performed to generate final predictive models. Receiver operator characteristic (ROC) curves were constructed and discrimination of models was assessed by comparing areas under the curve (AUC) with MedCalc^®^ 11.6.1. software [39]. All other statistical analysis was performed by IBM SPSS Statistics 20.

## Results

### Animal model of temporary middle cerebral artery occlusion (tMCAO)

**Fig 1:**
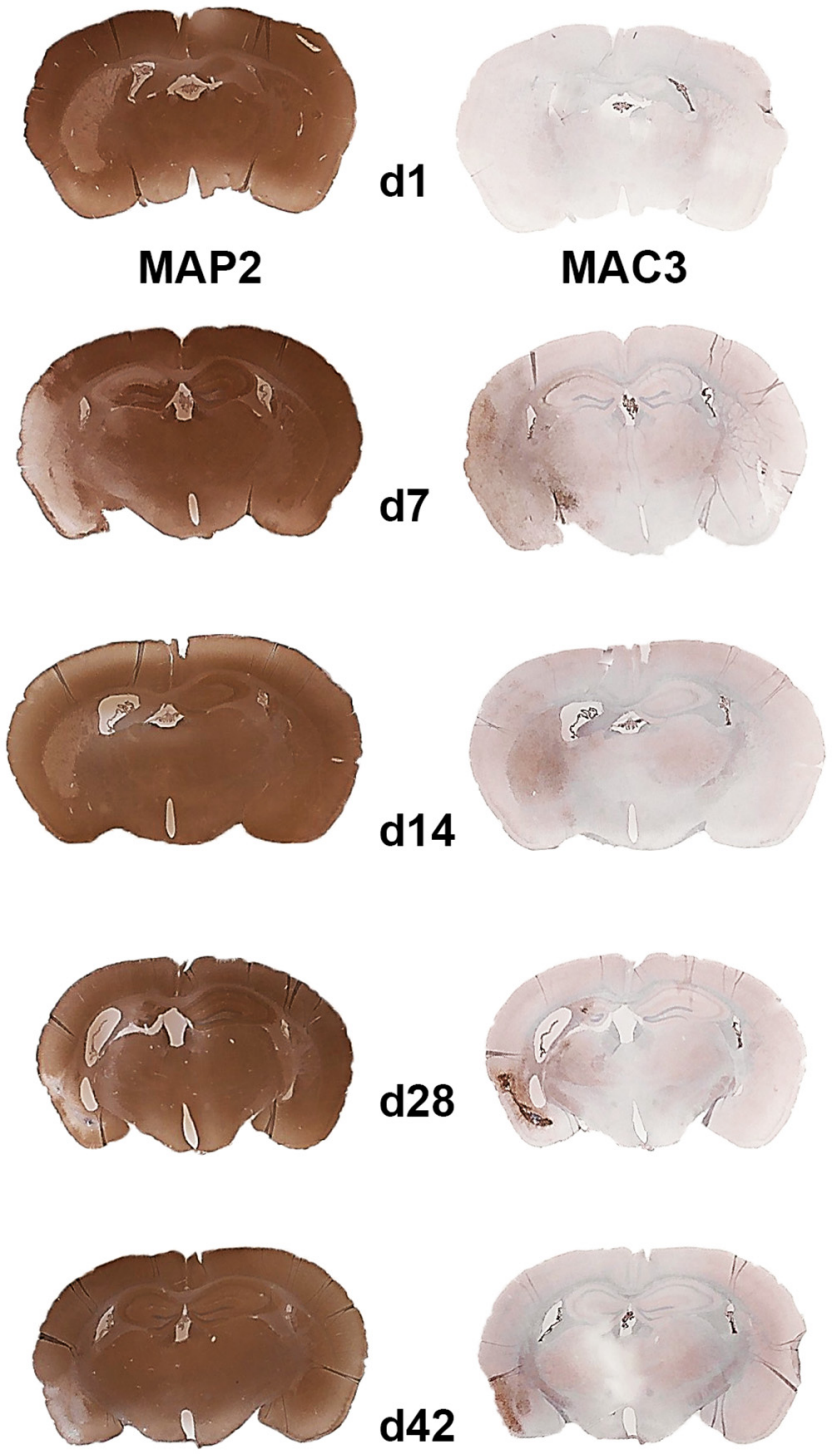
Immunohistochemistry in the mouse brain at days 1, 7, 14, 28 and 42 after temporal middle cerebral artery occlusion. Reduced MAP2 immunostaining delineates ischemic lesions (left column). Macrophage/microglia infiltration is shown by MAC3 immunostaining (right column).

**Fig 2:**
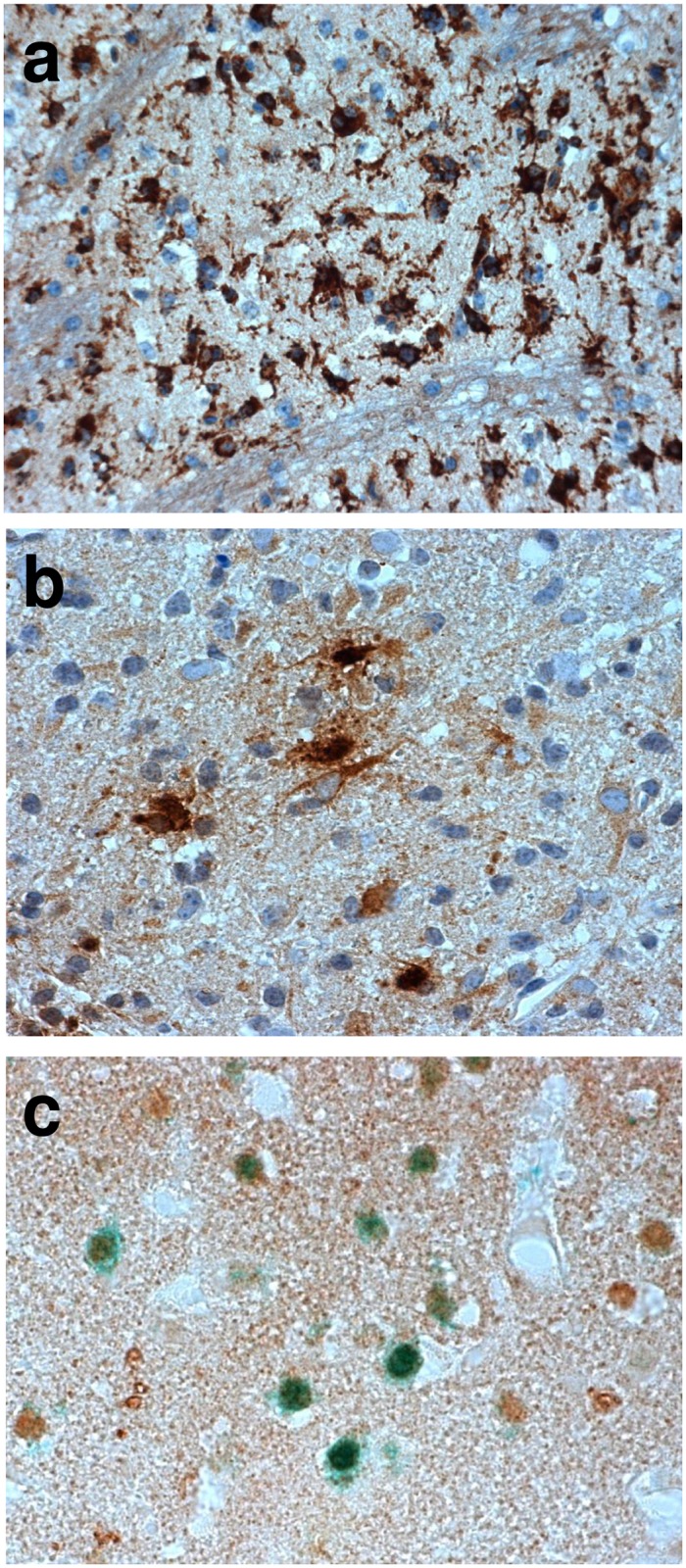
Immunohistochemistry in the mouse brain after temporal middle cerebral artery occlusion (tMCAO). Macrophage/microglia infiltration (anti-MAC3, brown, **a**) and lipocalin-2 (LCN2) expression in cells with macrophage/microglia and astrocyte morphology (anti-LCN2, brown, **b**) in chronic ischemic lesions. LCN2 immunoreactivity is also found in neurons in peri-infarct areas at day 7 after tMCAO (anti-LCN2, brown; anti-NeuN, green; **c**).

**Fig 3:**
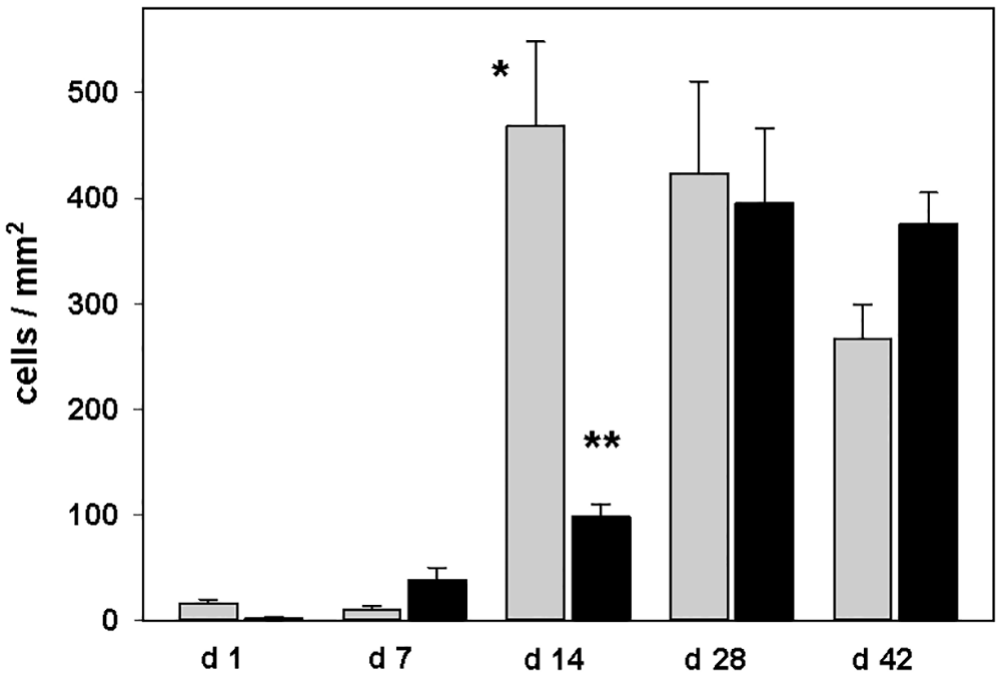
Quantification of lipocalin-2 positive cells (grey) and nonheme iron staining cells (black) in the ischemic striatum in mice after temporal middle cerebral artery occlusion (tMCAO). Data are presented as mean and standard error; *p<0.001 day 14 vs. days 1 and 7; **p<0.01 day 14 vs. day 7, p<0.001 day 14 vs. days 1, 28 and 42.

**Fig 4:**
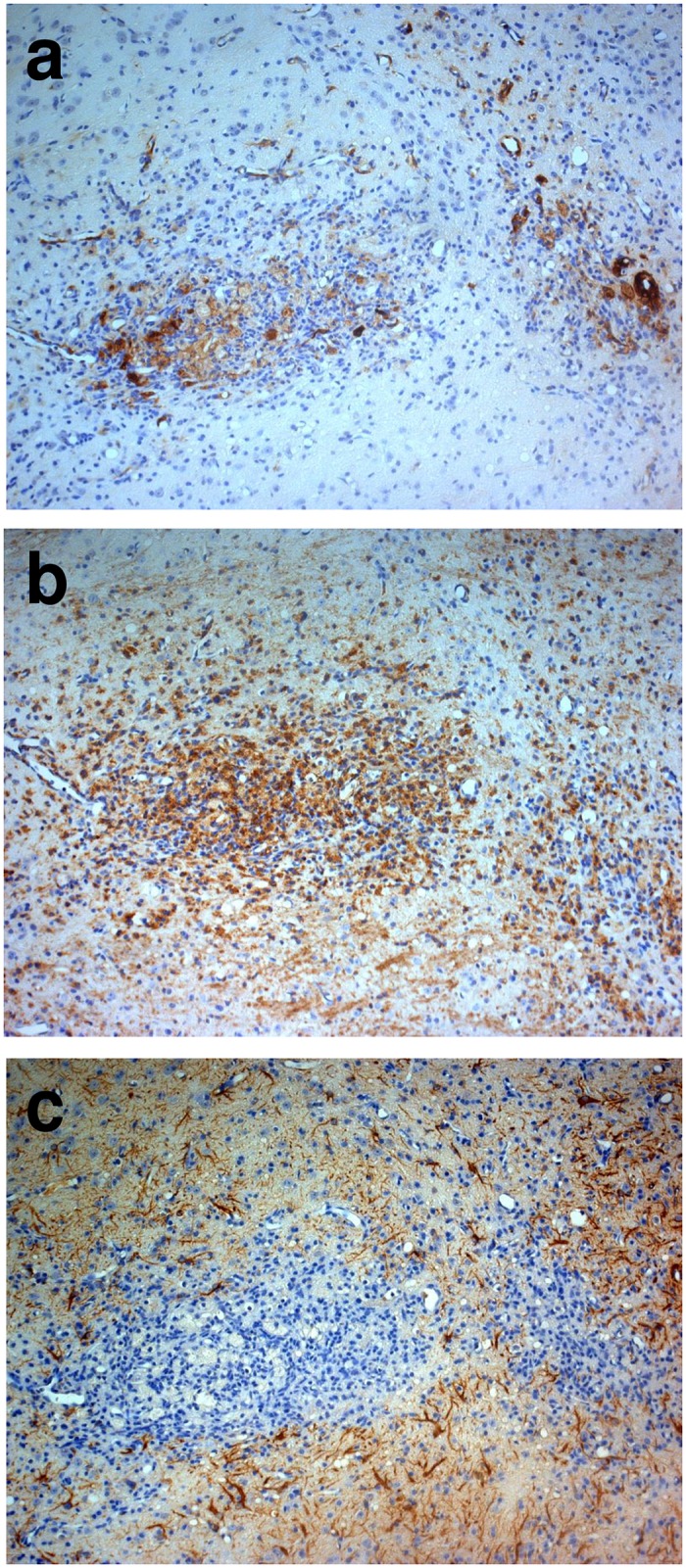
Immunohistochemistry in the mouse brain after temporal middle cerebral artery occlusion (tMCAO). In adjacent serial sections at day 14 following tMCAO, cellular nonheme iron staining (**a**) co-localizes with macrophage/microglia (**b**) but not with glial fibrillary acidic protein (GFAP) immunoreactivity (**c**).

Ischemic brain lesions were delineated by a reduced MAP2 staining in the striatum and frequently the neocortex in tMCAO mice (Fig 1). Prominent macrophage/microglia (MAC3) immunoreactivity throughout ischemic lesions was found (Figs 1 and 2a). LCN2 was expressed in cells with macrophage/microglia and astrocyte morphology in subacute and chronic ischemic lesions (Fig 2b). Single neurons in peri-infarct areas with LCN2-immunoreactivity 7 days post tMCAO were identified by double immunostaining for LCN2/NeuN (Fig 2c). The number of LCN2 expressing cells gradually increased from the first day post tMCAO with a maximum two weeks later (Fig 3; S1 File). Notably, the amount of LCN2 positive cells gradually decreased from that time-point until the end of the experiment (Fig 3; S1 File). No LCN2 positive cells were found in non-ischemic hemispheres at all analyzed time-points. In ischemic hemispheres, accumulation of cellular nonheme iron was detectable one week post tMCAO and continued to increase. Highest cellular nonheme iron loads were found at day 28 post tMCAO (Fig 3, S1 File). In adjacent serial sections, nonheme iron staining co-localized with macrophage/microglia but not with GFAP immunoreactivity (Fig 4). Serum levels of LCN2 in mice 7 days post tMCAO showed no difference compared to controls (378.5 vs. 346.0 ng/ml; p = 0.72; S1 File). Levels of LCN2 in mouse serum did not correspond to the volume of ischemic lesions (data not shown).

### Plasma levels of LCN2 in ischemic stroke patients and clinical outcome

Plasma samples were obtained from 46 consecutive patients at median of 7 days (range 5–9 days) after the onset of ischemic stroke. Demographics, clinical characteristics, cerebrovascular risk factors and stroke classification of patients are given in Table 1.

**Table 1:** Demographic data and clinical characteristics of patients.

	all patients	mRS 0–2	mRS 3–6	p
**n**	46	13	33	-
**median age; years (IQR)**	71 (63–79)	68 (54–74)	76 (63–79)	0.107
**female sex (n)**	47.8% (22)	53.8% (7)	45.5% (15)	0.987
**median NIHSS (IQR)**	11 (6–17)	6 (5–8)	13 (7–18)	0.002
**thrombolysis (n)**	26.1% (12)	38.5% (5)	21.2% (7)	0.276
**post-stroke infections (n)**	58.7% (27)	23.1% (3)	72.7% (24)	0.006
**mean eGFR (IQR)**	75,6 ml/min/1.7 (85,4–65,7)	69,6 ml/min/1.7 (80,6–58,7)	77,9 ml/min/1.7 (89,0–66,8)	0.269
**hypertension (n)**	84.8% (39)	100% (13)	78.8% (26)	0.166
**hypercholesterolemia(n)**	47.8% (22)	61.5% (8)	42.4% (14)	0.400
**diabetes mellitus (n)**	21.7% (10)	23.1% (3)	21.2% (7)	1.000
**atrial fibrillation (n)**	41.3% (19)	23.1% (3)	48.5% (16)	0.214
**hemorrhagic transformation (n)**	13.0% (6)	7.7% (1)	15.2% (5)	0.659
**TACS (n)**	41.3% (19)	7.7% (1)	54.5% (18)	0.010
**PACS (n)**	50.0% (23)	69.2% (9)	42.4% (14)	0.190
**POCS (n)**	4.3% (2)	15.4% (2)	0	0.075
**LACS (n)**	4.3% (2)	7.7% (1)	3.0% (1)	0.490
**supra-aortic atherosclerosis (n)**	21.7% (10)	7.7% (1)	27.3% (9)	0.240
**cardio-aortic embolism (n)**	52.2% (24)	46.2% (6)	54.5% (18)	0.853
**small artery occlusion (n)**	0	0	0	-
**other uncommon stroke causes (n)**	6.5% (3)	7.7% (1)	6.1% (2)	1.000
**undetermined stroke causes (n)**	19.6% (9)	38.5% (5)	12.1% (4)	0.092

IQR, interquartile range; NIHSS, National Institutes of Stroke Scale; eGFR, estimated glomerular filtration rate; TACS, total anterior circulation syndrome; PACS, partial anterior circulation syndrome; POCS, posterior circulation syndrome; LACS, lacunar syndrome

**Fig 5:**
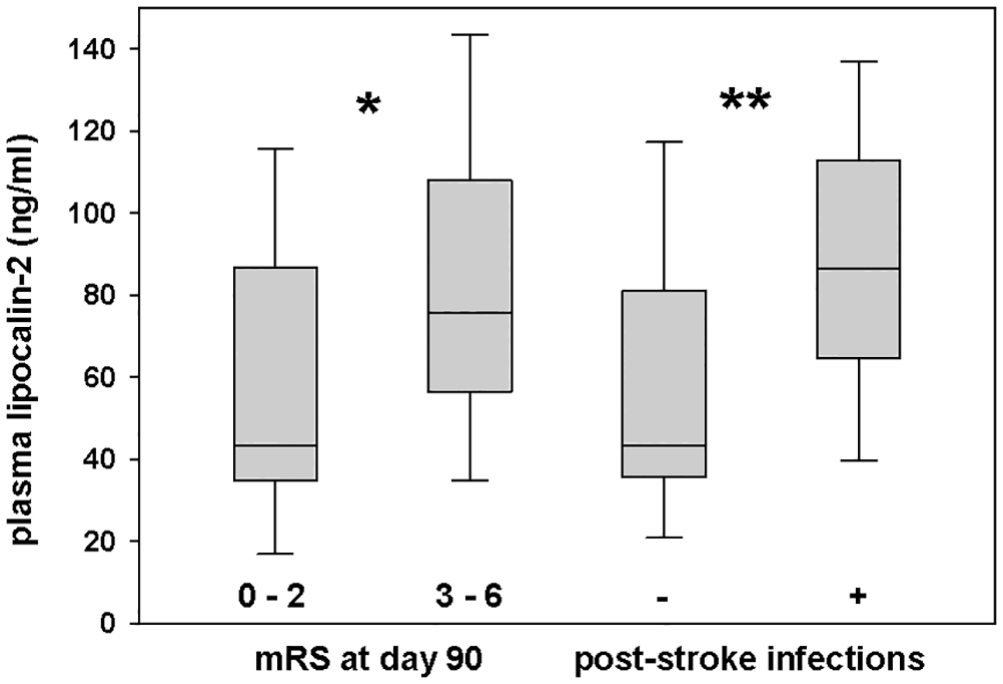
Plasma levels of lipocalin-2 in patients obtained at a median of 7 (range 5–9) days after ischemic stroke onset. First, patients were dichotomized into favourable (mRS 0–2) and unfavourable (mRS 3–6) outcomes assessed 90 days after stroke. Second, patients were dichotomized if they had developed post-stroke infections or not. Plots display the median, interquartile range (box), 10^th^ and 90^th^ percentiles (whiskers); *p<0.05; **p<0.01. Abbreviation: mRS = modified Rankin Scale

Notably, plasma levels of LCN2 did not correlate with NIHSS upon admission (r_S_ = 0.08; p = 0.58). A correlation of LCN2 plasma levels with the mRS at 90 days after stroke was found (r_S_ = 0.40; p<0.01). For further analysis, patients were dichotomized into favourable (mRS 0–2) and unfavourable (mRS 3–6) outcomes 90 days after stroke. Median NIHSS, the proportion of post-stroke infections and the proportion of total anterior circulation stroke (TACS) differed significantly between these patient subgroups (Table 1). In contrast to patients with mRS 0–2, those who had mRS 3–6 showed significantly higher median plasma levels of LCN2 measured one week after stroke (75.5 vs. 43.4 ng/ml; p = 0.03; Fig 5). 27 patients (58.7%) developed post-stroke infections: urinary tract infections (n = 17), pneumonia (n = 8), and other (n = 2). Among patients with post-stroke infections, the median plasma level of LCN2 was significantly higher as compared to patients with no infections (86.4 ng/ml vs. 43.4 ng/ml; p = 0.006; Fig 5, S2 File). Plasma LCN2 levels did not correlate with maximum CRP levels measured one week after stroke (r_S_ = 0.23; p = 0.12). Notably, CRP levels provided no prognostic information for clinical outcome 90 days post stroke (data not shown). Furthermore, no difference in LCN2 levels was found between patients with or without hypertension (data not shown). LCN2 plasma levels in our study are in the range of levels found previously in patients with acute ischemic or hemorrhagic stroke [14,15]. To our knowledge, there is no previous data about LCN2 levels measured one week after stroke onset as done in our study.

The relevance of LCN2 plasma levels to predict an unfavorable vs. favorable clinical outcome was evaluated by comparing predictive models. Model 1 included NIHSS, patients’ age, thrombolytic therapy and the estimated glomerular filtration rate (eGFR). The eGFR as a measure of chronic kidney disease is an independent predictor of poor outcome and long-term mortality in patients with stroke [40]. Previously, LCN2 was reported to negatively correlate with the eGFR [38] which was also found in our study (r_S_ = -0.321; p = 0.030). Predictive model 2 further included LCN2 (Table 2). The addition of LCN2 to the established prediction parameters significantly increased the AUC of generated ROC curves from 0.851 (model 1) to 0.935 (model 2), thereby demonstrating the value of LCN2 plasma levels measured one week after stroke for predicting clinical outcome at 90 days.

**Table 2:** Predictive models for clinical outcome 90 days after ischemic stroke.

model	variable	p	OR (95% CI)	AUC (95% CI)
1	NIHSS	0.011	1.433 (1.087–1.890)	0.851 (0.743–0.958)*
	age	0.067	1.108 (0.993–1.236)	
	thrombolysis	0.090	0.015 (0.015–1.344)	
	eGFR	0.202	1.038 (0.980–1.099)	
2	NIHSS	0.016	1.441 (1.071–1.939)	0.935 (0.864–1.000)*
	age	0.090	1.120 (0.982–1.277)	
	thrombolysis	0.096	0.096 (0.007–1.306)	
	eGFR	0.090	1.06 (0.991–1.134)	
	lipocalin-2	0.049	1.029 (1.000–1.059)	

*p = 0.048; OR, Odds ratio; CI, confidence interval; AUC, area under the curve; IQR, interquartile range; NIHSS, National Institutes of Stroke Scale; eGFR, estimated glomerular filtration rate

## Discussion

Our study is the first to describe long-term expression kinetics of LCN2 in tMCAO, an animal model of ischemic stroke. The presence of LCN2 in rat tMCAO and postmortem human ischemic brain was previously reported up to 3 days post stroke [22,41]. We could demonstrate a continuous presence of LCN2 in the ischemic mouse brain expressed predominantly by macrophages/microglia up to 42 days post tMCAO. Furthermore, we found neuronal LCN2-immunoreactivity in peri-infarct areas up to 1 week post tMCAO. Previous studies revealed a role of LCN2 in the migration of astrocytes, microglia and neurons by the induction of chemokine expression [24–26], thereby suggesting favorable effects of LCN2 in post-stroke remodeling. However, an association with the induction of astrogliosis and neuronal cell death has also been reported [23,26,27]. The deposition of nonprotein-bound iron after transient experimental ischemia as well as glial iron deposition 3–24 weeks after experimental ischemia has been found previously [42–44]. LCN2 has been shown to contribute to iron homeostasis. We observed an increase in the deposition of cellular nonheme iron in ischemic hemispheres with a decrease of LCN2 expression in our animal model. Comparable amounts of LCN2 were found in serum of tMCAO and control mice one week after ischemia. This suggests that a release of a significant amount of LCN2 from the ischemic brain into the circulation is unlikely. In this mouse model of tMCAO, all animals received preventive antibiotic treatment.

In ischemic stroke patients, higher plasma levels of LCN2 measured one week after stroke correlated with worse clinical outcome at 90 days in our study. Increased levels of LCN2 were associated with post-stroke infections and supposedly reflect the response of circulating neutrophils to infections. Neutrophils serve as a major reservoir of LCN2 [4–7]. The average lifespan of circulatory neutrophils is 5.4 days [45]. Therefore, the increase in peripheral LCN2 levels at one week after stroke rather derives from newly recruited neutrophils and not from a response to the acute ischemia at the day of stroke onset.

The strength of our study is the exact timing with a predefined interval from stroke onset to blood sampling to include information about changes of circulating LCN2 upon ongoing post-stroke infections. Previous studies about blood biomarkers in stroke patients have not applied such a strict criterion but rather lumped together sampling at the day of the acute incident and sampling at several days afterwards. In previous studies, the different timing of blood sampling has blurred a distinction whether interleukin-6 could serve as a marker of simultaneously evolving post-stroke infections or rather as their predictor [46]. In our study, with an interval of one week from stroke onset to blood sampling, we could show that circulating LCN2 is a marker of simultaneously evolving infections and contributes to the prediction of clinical outcome. Although our study cohort is selected, with our exclusion criteria we intended to exclude factors other than post-stroke infections which could have an influence on circulating LCN2.

## Conclusion

A wide range of biomarkers has been evaluated for their relevance as predictors of clinical outcome after ischemic stroke [46–49]. However, no infection-related serum/plasma biomarker is currently applied in clinical routine. Our study shows that LCN2, measured in peripheral blood one week after stroke onset, is an infection-related biomarker related to the clinical outcome at 90 days. Although LCN2 is expressed in the ischemic brain, peripherally circulating LCN2 is not a marker of brain damage. Biomarkers in peripheral blood which could improve diagnosis and treatment of post-stroke infections are needed. Therefore, large multicenter efforts should aim to replicate the findings of this study.

## Supporting Information

S1 File Immunohistochemistry raw data after temporal middle cerebral artery occlusion (tMCAO) in mice. Number of lipocalin-2 (LCN2) positive cells and nonheme iron staining cells in the ischemic striatum after tMCAO. Serum levels of LCN2 in mice 7 days post tMCAO.
(XLSX)

S2 File Raw data of ischemic stroke patients. (XLSX)
